# Characterization of the molecular dysfunctions occurring in Aicardi-Goutières syndrome patients with mutations in ADAR1

**DOI:** 10.1016/j.gendis.2023.05.020

**Published:** 2023-07-13

**Authors:** Sofian Al Wardat, Loredana Frassinelli, Elisa Orecchini, Federica Rey, Silvia Anna Ciafrè, Silvia Galardi, Jessica Garau, Stella Gagliardi, Simona Orcesi, Davide Tonduti, Stephana Carelli, Cristina Cereda, Ernesto Picardi, Alessandro Michienzi

**Affiliations:** a Department of Biomedicine and Prevention, University of Rome Tor Vergata, Rome 00133, Italy; b Pediatric Clinical Research Center “Romeo ed Enrica Invernizzi”, Department of Biomedical and Clinical Sciences, University of Milano, Milano 20157, Italy; c Center of Functional Genomics and Rare Diseases, Department of Pediatrics, Buzzi Children's Hospital, Milano 20154, Italy; d Department of Child Neurology and Psychiatry, IRCCS Mondino Foundation, Pavia 27100, Italy; e Unit of Pediatric Neurology, C.O.A.L.A (Center for Diagnosis and Treatment of Leukodystrophies), Buzzi Children's Hospital, Milano 20154, Italy; f Department of Biosciences, Biotechnologies and Environment, University of Bari “Aldo Moro”, Bari 70125, Italy; g Institute of Biomembranes, Bioenergetics and Molecular Biotechnologies (IBIOM), National Research Council (CNR), Bari 70126, Italy; h Biostructures and Biosystems National Institute (INBB), Rome 00136, Italy

Aicardi-Goutières syndrome (AGS) is a systemic inflammatory disorder caused by mutations in any one of the nine different genes, whose deficiency provokes a type I (interferon) IFN response probably central to pathogenesis.^1^ ADAR1, one of the genes mutated in AGS (AGS6), encodes for an enzyme that belongs to the ADAR family (ADAR1, ADAR2, and ADAR3) that catalyzes the conversion of adenosine to inosine within double-stranded RNAs (dsRNAs) (RNA editing A-to-I).^2^^,^^3^ Two main isoforms of ADAR1 are expressed in mammals: the full-length p150 that is interferon-inducible and the constitutively expressed shorter isoform p110.^2^^,^^3^

Since inosines are recognized as guanosines by most cellular machinery, RNA editing mediated by ADARs occurring in the protein-coding sequence can lead to the formation of an altered protein (recoding). Indeed, most RNA editing occurs in non-coding sequences (including Alu sequences) affecting different aspects of the RNA metabolism.^2^ It has been proposed that the ADAR1 p110 isoform exerts an anti-apoptotic role,^2^ whereas the p150 isoform plays a role as a suppressor of the IFN signaling and response.^3^ In particular, the p150 isoform marks through RNA editing the endogenous “self” dsRNAs, thus avoiding their improper recognition by the cytosolic dsRNA receptor MDA5 that activates innate immune pro-inflammatory responses.^3^ The molecular mechanisms linking ADAR1 mutations with the AGS pathogenesis are still unclear.

As immortalized patient-derived lymphoblastoid cell lines (LCLs) were previously successfully used as AGS *in vitro* models, we employed four AGS6-LCLs with different mutations in ADAR1 (Fig. 1A), most of which led to amino acid substitutions in domains critical for the enzyme activity. By Western blotting analysis, we showed the decrease of both ADAR1 isoforms in AGS6 LCLs compared to their levels in control LCLs (Fig. S1A). Thus, some of the AGS6 mutations may affect protein stability/expression. This result was further confirmed in fibroblasts isolated from the skin biopsy of a patient carrying the Gly1007Arg mutation (Fig. S1B).

**Figure 1:**
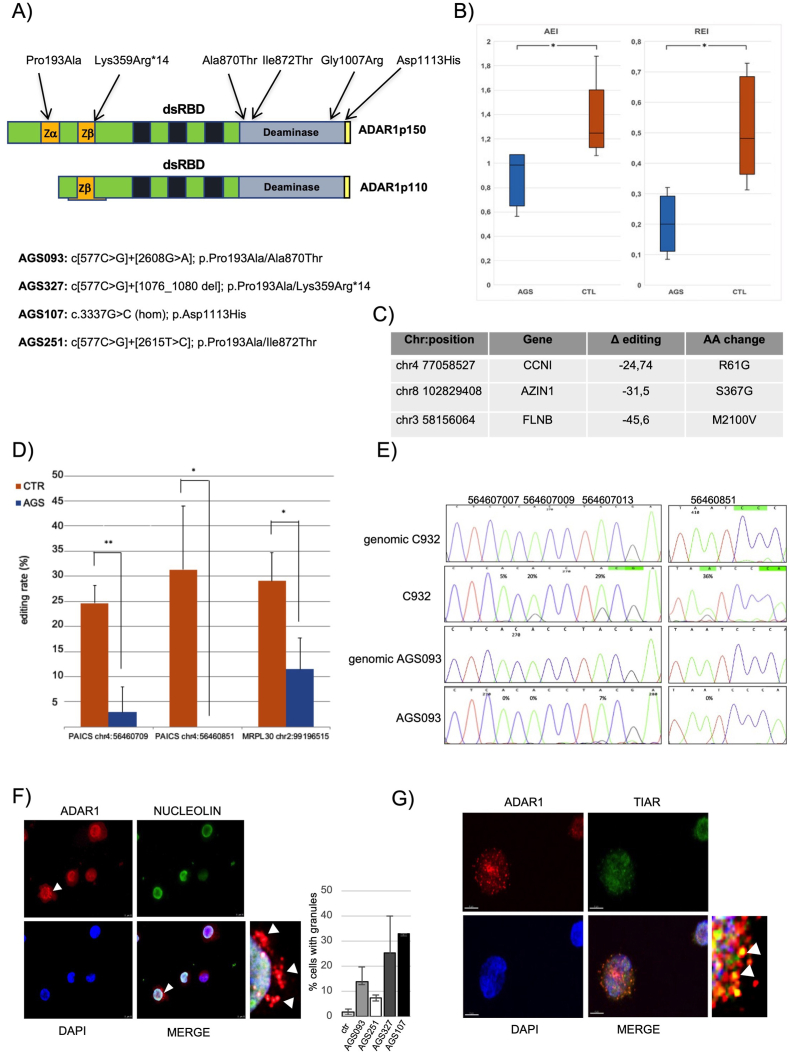
ADAR1 mutations affect enzyme activity and subcellular localization in AGS6 patient-derived cells. **(A)** Schematic representation of the p150 and p110 isoforms of ADAR1. The deaminase domain, the dsRNA binding domains (dsRDBs), and the Z domains are shown. Moreover, the positions of the mutations within the nucleotide sequence and the position of the corresponding amino acid change in the ADAR1 p150 isoform found in the AGS6-LCLs and AGS6-fibroblasts employed are indicated. **(B)** RNA editing analysis of the RNA-seq data. Left panel, Alu editing index (AEI) distributions (box plot, median); right panel, recoding editing index (REI) value distributions (box plot, median). Two-tailed Mann–Whitney U test was applied (*P* ≤ 0.05). **(C)** RNA editing analysis of the RNA-seq data. Statistically significant differential recoding sites. For each editing site, we reported the chromosomal position, target gene, the difference between mean editing levels of AGS6 and control samples, and amino-acid changes induced by RNA editing. Negative Δs are associated with lower editing in AGS6 LCLs than in control LCLs **(D)** RNA editing levels of two sites in the PAICS and one in the MRPL30 transcripts in AGS6 and control LCLs. The *X*-axis indicates the transcript name and the chromosomal position of the editing site. The *Y*-axis indicates the editing percentage, determined by RT-PCR followed by DNA Sanger sequencing. Inosine is read as guanosine during reverse transcription; therefore, any A-to-I change in the target RNA appears as an A-to-G change in the resulting PCR products. *n* = 3; the *P*-value was calculated by a student's *t*-test and is indicated above each histogram (∗*P* < 0.05, ∗∗*P* < 0.01). **(E)** Representative electropherograms showing the RNA editing A-to-I of specific adenosines (the chromosome position is indicated) within the PAICS transcript in AGS6 LCLs, control LCLs, and the corresponding genomic DNA. Total RNA and genomic DNA isolated from the cells were subjected to RT-PCR and PCR amplification respectively with a specific set of primers followed by DNA Sanger sequencing. The green peak represents the unedited (A) site, and the black peak represents the edited (G) site. The percentage of editing efficiency is indicated. **(F)** Subcellular localization of ADAR1 variants. AGS6-LCLs (AGS327) were stained with either an anti-ADAR1 (red) or anti-Nucleolin (green) antibody and DAPI (blue) to visualize the nuclei. A high-magnification image of the ADAR1-containing granules is shown. The percentage of the LCLs containing granules in three independent immunofluorescence experiments is reported on the right. **(G)** AGS6-LCLs (AGS327) were stained with either an anti-ADAR1 (red) or anti-TIAR (green) antibody and DAPI (blue) to visualize the nuclei. A high-magnification image of the regions containing the ADAR1 and TIAR-containing granules is shown.

To investigate the impact of ADAR1 mutations on global gene expression and RNA editing, we analyzed the AGS6 LCL transcriptome. Whole-transcriptome RNA-seq of AGS6 and control LCLs was performed by Illumina next-generation sequencing. By using a significance level of Padj ≤ 0.05 and |Log2fold change| > 1.5, we found a total of 725 differentially expressed genes (DEGs) in AGS6 LCLs compared to controls, of which 362 were up-regulated and 363 down-regulated (Table S1). DEGs were subjected to principal component analysis (Fig. S2A) and different ontology sources were used to perform enrichment analysis as reported in Figure S2B–D. When compared to the background dataset, DEGs presented a different 3′UTR length (Fig. S2E), thus possibly affecting RNA metabolism. Of note, a significant fraction of the DEGs in AGS6 LCLs encodes for lncRNAs (218 out 725, 35 up-regulated and 183 down-regulated), a class of RNAs that regulate gene expression at different levels in various diseases. By applying specific tools (MATS), we found hundreds of transcripts that are differentially spliced in AGS6 LCLs compared to control LCLs (Table S2). This result was expected since a role of ADAR1 in splicing regulation was reported.^2^^,^^3^

It was previously shown that ADAR enzymes may affect microRNA biogenesis or their target selection,^2^^,^^3^ thus we carried out profiling of microRNAs. Twenty-seven differentially expressed (DE) microRNAs were found in AGS6-LCLs compared to controls with a *P* value ≤ 0.06 (Table S3). The expression of miR-320c and miR-181a-2-3p was validated by RT-qPCR assays (Fig. S3A). Previous *in vitro* studies showed that the p150 isoforms of some of the ADAR1 variants expressed in AGS6 patients are impaired in their catalytic activity.^4^ We assayed RNA editing by performing RNA-seq experiments as described above. RNA editing was profiled using the REDItools suite, and the Alu editing index (AEI) and recoding index (REI) which are relevant metrics to measure the RNA editing activity, in Alu sequences or at protein-coding regions, respectively, were calculated. As expected, we found that both indexes were decreased in AGS6 samples compared to controls (Fig. 1B), indicating that the global RNA editing activity is affected in AGS6. Several statistically significant differentially edited sites were found in AGS6 LCLs (Table S4), many of which were in Alu sequences, and a few within protein-coding sequences (Fig. 1C). We validated the RNA editing reduction in 8 editing sites by RT-PCR followed by DNA Sanger sequencing (Fig. S3B). In three of them (Fig. 1D, E), this reduction was statistically significant. RNA editing reduction was confirmed also in AGS6 fibroblasts (carrying the pGly1007Arg mutation) (Fig. S3C). By using specific bioinformatic tools, we identified 60 long dsRNAs embedded within mature mRNAs that were significantly less edited in AGS6 LCLs compared to control LCLs (Padj <0.05; Table S5, 6). Interestingly, 13 out of 60 of these long dsRNA-containing transcripts were previously reported among the most efficient MDA5 ligands (*i.e.*, VHL and PSMB2), suggesting their possible immunogenicity. These results are of paramount importance since they provide the first evidence of an overall alteration of the RNA editing mediated by ADAR1 in patient-derived cells.

To test whether ADAR1 mutations causing AGS6 may lead to a mislocalization of the two deaminase isoforms, we performed immunofluorescence assays in the different LCLs. This showed that ADAR1 is mostly localized in the nucleus as expected due to the higher expression level of the p110 nuclear isoform compared to the cytoplasmic p150 isoform (Fig. S1A, 4).^2^^,^^3^ Strikingly, in the AGS6 LCLs, we found that ADAR1 forms cytoplasmic granules in about 8%–33% of the examined cells (Fig. 1F, white arrows). This phenotype is more evident in the AGS107-LCLs and less in AGS93-LCLs, indicating that the assembly of these granules may depend on the specific variant expressed. In contrast, less than 2% of the examined control LCLs showed similar granules. As shown in Figure 1G, TIAR, a marker of stress granules (SGs), co-localizes with some of these granules. SGs are RNP granules that form as a cellular response to different stresses.^5^ Notably, the LCLs analyzed were not previously treated with any type of stressor known to induce SGs. Transient transfection of HeLa cells with plasmids expressing two of the ADAR1 p150 variants (Pro193Ala and Ala870Thr) harbored in some of the AGS6 LCLs (Fig. 1A), led to the formation of TIAR-containing granules (SG-like), most of which overlap with ADAR1 immunofluorescence signal (Fig. S5A, B). This result confirms that missense mutations in ADAR1 partially disrupt normal enzyme localization thus leading to the formation of cytoplasmic granules, some of which resemble SGs (Fig. 1G). By using an anti-MDA5 antibody, we also showed co-localization between ADAR1 and MDA5 in some of these granules (Fig. S6A). This co-localization is not observed in control LCLs (data not shown). It is worth noting that in 75% of the AGS6 LCLs showing the ADAR1-containing granules, the co-localization with either MDA5 or TIAR proteins can be observed in some of these granules. By performing a UV crosslinking immunoprecipitation (CLIP) experiment with an anti-MDA5 antibody using total cell extracts, we showed that MDA5 co-immunoprecipitates with both ADAR1 isoforms in control and AGS6 LCLs (Fig. S6B). Thus, the physical association between ADAR1 and MDA5 occurs independently from granule formation.

Overall, our results clearly showed that mutations in ADAR1 deeply affect gene expression of AGS6 patient-derived cells at different layers: non-coding RNA expression, global RNA editing, alternative splicing, 3′UTR length, and cytoplasmic granule formation of unknown function.

## Author contributions

Alessandro Michienzi contributed to the conception and design of the research. Silvia Anna Ciafrè contributed to the research design and helped in writing the manuscript. Stephana Carelli and Cristina Cereda participated in making the experimental plan, interpreting the data, and revising the manuscript. Federica Rey isolated primary fibroblasts and performed some data analysis. Jessica Garau, Stella Gagliardi, Elisa Orecchini, and Silvia Galardi were involved in the investigation. Davide Tonduti, Simona Orcesi partecipated in data discussion. Loredana Frassinelli and Sofian Al Wardat performed most of the molecular, cellular, and biochemistry experiments. Ernesto Picardi helped in RNAseq data analysis and RNA editing profiling. All appropriate contributors were listed as authors and all authors agreed to the manuscript's content and its submission to *Genes & Diseases*.

## Conflict of interests

The authors declare no conflict of interests.

## Funding

This work was supported by the grants from United Leukodystrophy Foundation (to Alessandro Michienzi) and partially supported by the 10.13039/501100003196Italian Ministry of Health (No. GR-2019-12368701 to Davide Tonduti and Cristina Cereda).

## Figures and Tables

**figs1:**
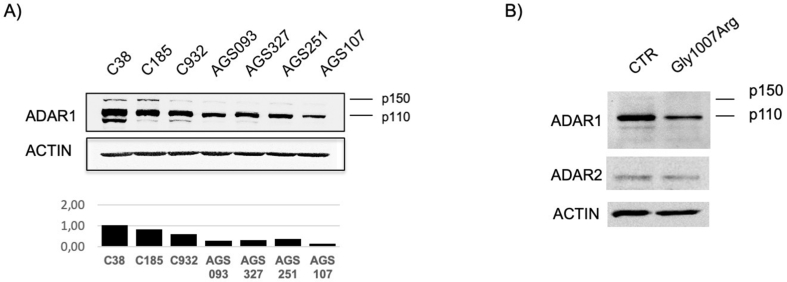
Intracellular levels of ADAR1 in patient-derived cells. A) Western Blot analysis of total cell extracts prepared from the control LCLs and AGS6 LCLs using antibodies anti-ADAR1 and anti-Actin. The level of ADAR1 p110 isoform in the different samples is reported in the bottom diagram. The X-axis indicates the cell line name. The Y-axis indicates p110 level. The level in sample C38 is set as = 1. The ADAR1 p150 follows the same trend. B) Western Blot analysis of total cell extracts prepared from the fibroblasts isolated either from the skin of an AGS6 patient carrying the pGly1007Arg mutation or from a healthy control using antibodies anti-ADAR1, -ADAR2 and anti-Actin.

**figs2:**
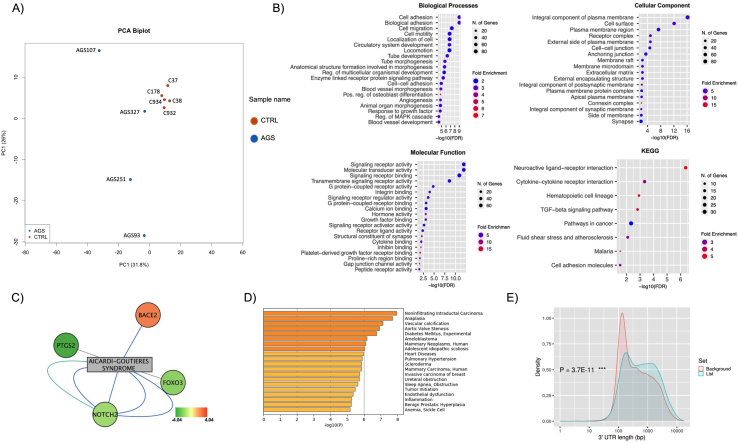
Gene enrichment analysis of the RNA-seq data. A) PCA plots of RNA-seq data (based on the top 500 most variable genes). Each blue dot indicates one AGS6 sample, and each orange dot indicates one control LCL sample B) Enrichment analysis was carried out with the following ontology sources: GO Biological Processes, GO Cellular Localization, GO Molecular Function and KEGG. C) and D) Enrichment analysis carried out with DisGeNET. Bar graph of enriched terms across input gene lists, colored by p-values. E) 3’UTR Length as obtained with the ShinyGo webtool.

**figs3:**
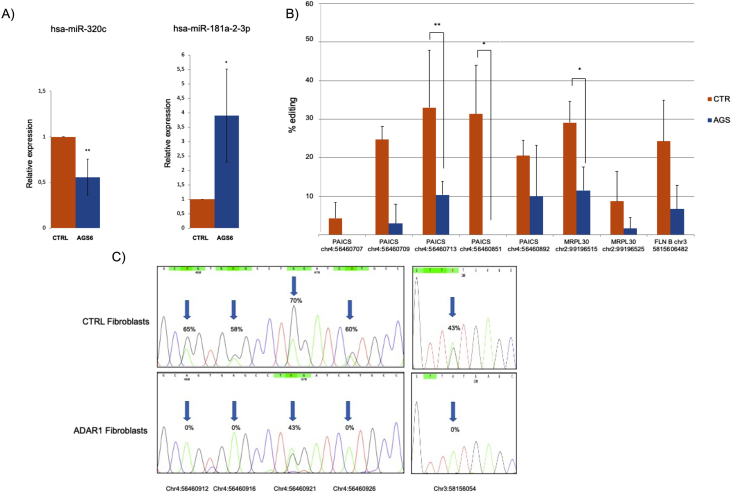
Validation of miRNA expression and RNA editing. A) Quantification of hsa-mir-320c and hsa-miR-181a-2-3p by RT-qPCR. Data are shown as means +/- SD from 3 AGS6 LCLs and 3 control LCLs samples. P values were calculated by two-tailed t test and are indicated above each histogram (∗P<0.05, ∗∗P<0.01). B) Validation of the RNA editing sites in LCLs. The RNA editing efficiency of 8 editing sites was measured by RT-PCR followed by DNA Sanger sequencing in three AGS6 and three control LCLs. The X-axis indicates the transcript name and the chromosomal position of the editing site. The Y-axis indicates the editing percentage; the p-value was calculated by a student’s t test (∗P<0.05, ∗∗P<0.01). C) Validation of the RNA editing sites in fibroblasts. Representative electropherograms showing the RNA editing A-to-I of specific adenosines (the chromosome position is indicated) within the PAICS and FlnB transcripts in AGS6 (pGly1007Arg) or control fibroblasts. Total RNA isolated from the cells was subjected to RT-PCR with a specific set of primers followed by DNA Sanger sequencing. The green peak represents the unedited (A) site, and the black peak represents the edited (G) site. The percentage of editing efficiency is indicated.

**figs4:**
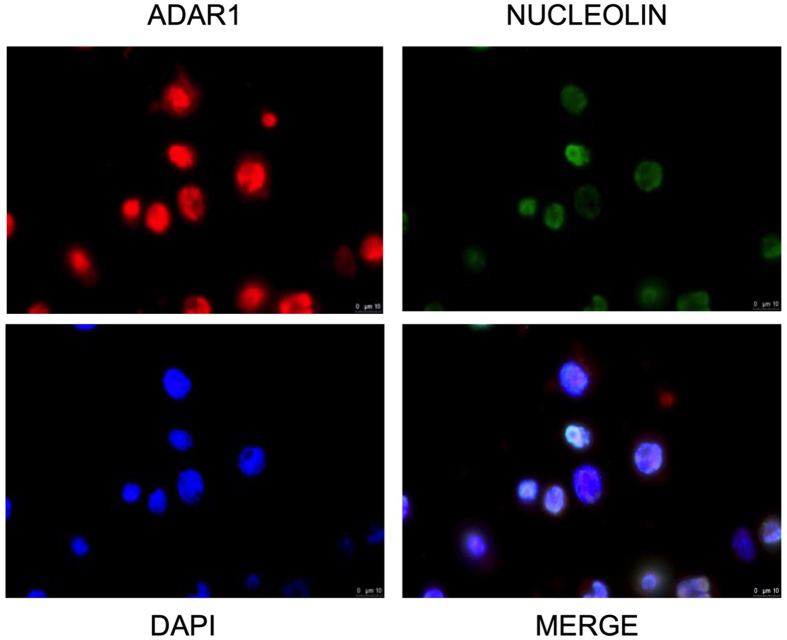
Subcellular localization of ADAR1 wt Control LCLs (C38) were stained with either an anti-ADAR1 (red) or anti-Nucleolin (green) antibody and DAPI (blue) to visualize the nuclei.

**figs5:**
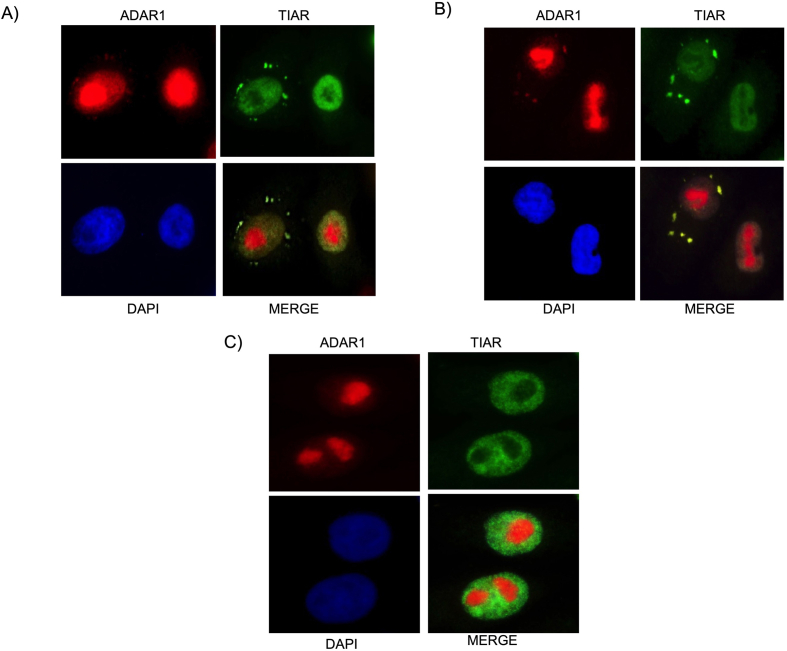
Subcellular localization of the ADAR1 variants overexpressed in HeLa cells. HeLa cells were transiently transfected with either vectors expressing the p150 wt ADAR1 protein (panel C) or vectors expressing some of the p150 variants, such as pADAR1 870 (expressing the pAla870Thr variant, panel A) and pADAR1 193 (expressing the pPro193Ala variant, panel B). Transfected cells were analyzed by immunofluorescence. In particular, cells were stained with either an anti-ADAR1 (red) or anti-TIAR (green) antibody and DAPI (blue) to visualize the nuclei.

**figs6:**
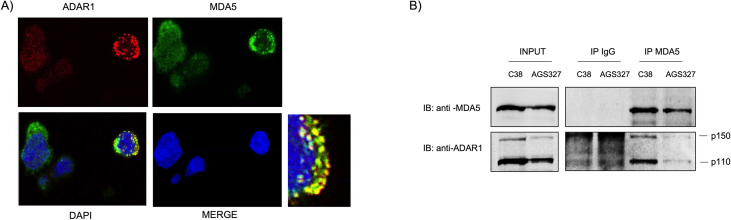
Interaction between ADAR1 and the cytosolic dsRNA sensor MDA5. A) AGS6-LCLs (AGS327) were stained with either an anti-ADAR1 (red) or anti-MDA5 (green) antibody and DAPI (blue) to visualize the nuclei. A high magnification image of the region containing the ADAR1, and MDA5-containing granules is shown. B) Lysates of AGS6 and control LCLs were UV Cross-Linked and Immunoprecipitated with an anti-MDA5 antibody (IP MDA5) or with control IgGs (IP IgG) followed by WB analysis with anti-MDA5 or anti-ADAR1 antibodies. WB analysis of 10 μg of LCLs lysate inputs is shown.

## Appendix A Supplementary data

Table S1 List of gene expression values calculated by DESeq2. For each gene we report the mean of normalized counts for all samples (baseMean), the log2 fold change: affected vs control (log2FoldChange), the standard error: affected vs control (lfcSE), the Wald statistic: affected vs control (stat), the Wald test p-value: affected vs control (pvalue) and the BH adjusted p-value (padj). In addition, we also report the gene type (lncRNA or protein-coding) and the normalized counts for each sample (AGS107, AGS251, AGS327, AGS93 for affected and C178, C37, C38, C932, C934 for controls). Table S1

Table S2 Output tables of the rMATS software for the identification of deregulated alternative splicing events. Each alternative splicing event type is reported in an independent spreadsheet: SE.MATS. JC for skipped exons, MXE.MATS.JC for mutually exclusive exons, A3SS.MATS.JC for alternative 3' splice sites, A5SS.MATS.JC for alternative 5' splice site and RI.MATS.JC for retained introns. All spreadsheets share the following columns: ID (rMATS event id), GeneID (Gene id), geneSymbol (Gene name), chr (Chromosome), strand (Strand of the gene), IJC_SAMPLE_1 (Inclusion counts for affected), SJC_SAMPLE_1 (Skipping counts for affected), IJC_SAMPLE_2 (Inclusion counts for controls), SJC_SAMPLE_2 (Skipping counts for controls), IncFormLen (Length of inclusion form, used for normalization), SkipFormLen (Length of skipping form, used for normalization), PValue (Significance of splicing difference between the two sample groups), FDR (False Discovery Rate calculated from p-value), IncLevel1 (Inclusion level for affected), IncLevel2 (Inclusion level for controls), IncLevelDifference (average(IncLevel1) - average(IncLevel2)). Spreadsheets specific counts are described in the rMATS documentation freely available at the GitHub page https://github.com/Xinglab/rmats-turbo/blob/v4.1.2/README.md). Table S2

Table S3 Top 27 differentially expressed microRNAs in AGS6 LCLs. Table S3

Table S4 Differential RNA editing analysis for sites detected in Alu elements (ALU), repetitive non Alu elements (REP NON ALU) and non-repetitive regions (NON REP). For each spreadsheet, we report the genomic location (according to hg38 genome assembly), the reference and alternative nucleotide, the strand, the P-value (according to REDIT tool), a flag indicating if the site is significant or not, the editing levels in AGS and Controls samples, the name of repetitive element and its family, the gene location (detected by ANNOVAR according to GENCODE V28) and the gene name (detected by ANNOVAR according to GENCODE V28).Additionally, we provide the list of recoding editing sites (genomic coordinates according to hg38 genome assembly) and the corresponding editing levels in AGS and Control samples. Table S4

Table S5 RNA editing analysis in individual long double strands (longDS) detected in mature transcripts (the longest isoform has been used) as described in the Methods section. Each column reports: the coordinates of the DS rna (dsRNA_1 for first strand and dsRNA_2 for second strand) according to hg38 assembly, the length of the DS RNA (LenDsRNA), the transcript (TranscriptName) and gene name (GeneName), the repeated elements in the DS RNA (RepeatsInDsRNA_1 and RepeatsInDsRNA_2), the AEI-like index for each sample, the mean AEI-like value for each group of samples (Mean-AGS for affected and Mean-CTRL for controls), the difference between mean AEI-like values (delta), the t-test statistics (Test), the t-test p-value (Pval), the BH corrected p-value (Qval) and a flag (yes or no) indicating the significance (IsSig). Table S5

Table S6 RNA editing analysis in all longDSs detected in mature transcripts. Each column reports: the gene (GeneName) and transcript (TranscriptName) name, the AEI-like index for each sample, the t-test statistics (Test), the t-test p-value (Pval), the BH corrected p-value (Qval) and a flag (yes or no) indicating the significance (IsSig). Table S6
