# Supplementary material for: Characterization of the molecular dysfunctions occurring in Aicardi-Goutières syndrome patients with mutations in ADAR1
Source: Genes Dis. 2023 Jul 13;11(3):101028. doi: 10.1016/j.gendis.2023.05.020 (PMC10827400; doi:10.1016/j.gendis.2023.05.020)
Supplement: Table S3 — Top 27 differentially expressed microRNAs in AGS6 LCLs. [file mmc3.docx]

| **microRNA name** | **Log2 fold change-AGS/ctrl** | **P value** |
| --- | --- | --- |
| **hsa-miR-551b-3p** | 2,658166227 | 0,001462332 |
| **hsa-miR-1254** | 2,320017454 | 0,011560388 |
| **hsa-miR-151a-5p** | 1,878656181 | 0,015488605 |
| **hsa-miR-3661** | 1,751782755 | 0,015551613 |
| **hsa-miR-152-5p** | 1,741829025 | 0,016142701 |
| **hsa-miR-25-5p** | 1,732692055 | 0,019947002 |
| **hsa-miR-3200-3p** | 1,462471792 | 0,02071229 |
| **hsa-miR-222-3p** | 1,369030499 | 0,022308781 |
| **hsa-miR-3614-5p** | 1,333105318 | 0,023419375 |
| **hsa-miR-221-3p** | 1,206756388 | 0,024346563 |
| **hsa-miR-423-5p** | 0,918603316 | 0,025157498 |
| **hsa-miR-361-3p** | 0,888206514 | 0,027514665 |
| **hsa-miR-1248** | -1,322377644 | 0,036273953 |
| **hsa-miR-320d** | -1,333406862 | 0,037790951 |
| **hsa-miR-24-3p** | -1,419599908 | 0,038881033 |
| **hsa-miR-143-3p** | -1,56676934 | 0,03905276 |
| **hsa-miR-3940-3p** | -1,867118279 | 0,043091238 |
| **hsa-miR-1255a** | -2,040290805 | 0,045416085 |
| **hsa-miR-340-5p** | -2,435899044 | 0,049984528 |
| **hsa-miR-181a-3p** | 1,24488901 | 0,051625036 |
| **hsa-miR-1307-3p** | 0,980595106 | 0,054005256 |
| **hsa-miR-766-5p** | 2,679642932 | 0,054260969 |
| **hsa-miR-181a-2-3p** | 1,36147215 | 0,057785285 |
| **hsa-miR-221-5p** | 1,284858183 | 0,05990521 |
| **hsa-miR-151b** | 1,930928348 | 0,059977945 |
| **hsa-miR-320c** | -1,233100546 | 0,060013274 |

Top 27 differentially expressed microRNAs in AGS6 LCLs
