# Supplementary material for: Characterization of the molecular dysfunctions occurring in Aicardi-Goutières syndrome patients with mutations in ADAR1
Source: Genes Dis. 2023 Jul 13;11(3):101028. doi: 10.1016/j.gendis.2023.05.020 (PMC10827400; doi:10.1016/j.gendis.2023.05.020)
Supplement: Supplementary Materials and Methods [file mmc7.docx]

Supplementary Materials and Methods

Generation of LCLs

LCLs were obtained by EBV immortalization of peripheral blood mononuclear cells (PBMCs) isolated from either AGS6 patients or healthy controls by Doctor Chiara Baldo at the Human genetic laboratory, IRCCS Istituto Giannina Gaslini, Genoa, Italy. Cell lines were grown in RPMI 1640 medium (CARLO ERBA Reagents S.r.l., Cornaredo, Italy), supplemented with 20% fetal bovine serum (FBS) (CARLO ERBA Reagents S.r.l., Cornaredo, Italy), 0.3 mg/L L-glutamine, and 5% penicillin-streptomycin (CARLO ERBA Reagents S.r.l., Cornaredo, Italy) at 37 °C in a humidified atmosphere with 5% of CO2. Centrifugation was used to pellet cells, which were then washed in Phosphate-Buffered

Saline (PBS) and treated as needed

Fibroblasts isolation

Patients were enrolled at the COALA center of “V. Buzzi” Hospital in Milan (CE Approval Nr. Prot 4702/2018 amended in 2020). After signing an informed consent form, a punch skin biopsy of approximately 0.3×0.3×0.3 cm^3^ was obtained. Skin samples were washed twice with PBS and cut into 2 pieces using a sterile blade. The biopsy was placed into a 48-well plate and maintained in culture with DMEM High Glucose supplemented with 20% FBS, 1% penicillin/streptomycin and 1% L-glutamine, until fibroblasts were generated (approximately two weeks).

Cells maintenance

Lymphoblastoid cell lines (LCLs) were grown at 37°C in a humidified incubator and in an atmosphere of 5% CO2, in RPMI-1640, supplemented with 20% fetal calf serum, 50 U/ml penicillin, 50 mg/ml of streptomycin and 2 mM L-glutamine.

HeLa cells were grown at 37°C in a humidified incubator and in an atmosphere of 5% CO_2_, in Dulbecco’s Eagle’s Medium supplemented with 10% foetal calf serum, 50 U/ml penicillin, 50 mg/ml of streptomycin and 2 mM L-glutamine.

Primary fibroblasts were maintained in Dulbecco’s Eagle’s Medium supplemented with 20% fetal calf serum, 50 U/ml penicillin, 50 mg/ml of streptomycin and 2 mM L-glutamine.

RNAseq and data analysis

Total RNA was isolated from four AGS6 LCLs and five control LCLs by using mirVANA Isolation Kit (Thermo Fisher Scientific) and the quality assessed by gel-electrophoresis.

Directional RNA-Seq libraries were prepared from 150 ng of total RNA using the TruSeq Stranded Total RNA Sample Prep Kit (Illumina, San Diego, CA) according to the manufacturer’s protocol.

The Illumina RiboZero rRNA Depletion Kit was used to remove rRNA. Qualities of sequencing libraries were assessed with D1000 ScreenTape Assay using the 4200 TapeStation System (Agilent, Santa Clara, CA, USA) and quantified with Qubit™ dsDNA HS Assay Kit (Thermo Fisher Scientific).

Sequencing was performed on an Illumina NextSeq 500 platform (Illumina, San Diego, CA). Raw reads were quality-checked by FASTQC and trimmed by FASTP to remove adaptor sequences and low-quality regions (phred score cut off of 25). Cleaned reads were aligned onto the human genome (assembly GRCh38) by STAR providing known gene annotations from GENCODEv31. Read counts per gene were obtained by means of FeatureCounts taking into account their strand orientation (using the -s2 parameter).^6^ Raw data are freely available at SRA under the BioProject accession PRJNA937589. Differential expression analysis was performed using R package DESeq2 and only genes with |log2FC | ≥ 1 and a False Discovery Rate (FDR) ≤ 0.05 were retained for further analysis.^7^

The co-expression analysis between coding RNAs and ncRNAs was performed using Weighted gene co-expression network analysis (WGCNA) R package (<https://CRAN.R-project.org/package=WGCNA>).^8^ The soft thresholding power was chosen considering the criterion of approximate scale-free topology.

MicroRNA sequencing and analysis

The miRNome profiling was performed using the small RNAs fraction isolated from the LCLs (4 AGS6 LCL lines and 4 control LCLs) by the mirVana kit (Thermo Fisher Scientific). Indexed cDNA libraries were prepared using the TruSeq small RNA sample Preparation kit (Illumina, San Diego, CA) according to the manufacturer’s protocol and recommendations. Single end sequencing (1 × 50b), after fluorometric quantification, was performed on an Illumina NextSeq 500 platform.

Raw sequence data were processed to remove adapters from inserts of between 14 and 38 nt in length and subsequently mapped onto the human genome using Bowtie.^9^ Counts of reads exactly matching human miRNAs and (where annotated in miRBase) miRNA* sequences were submitted to the DESeq2 software for statistical analysis of differential expression using default parameters.^9^

RNA editing profiling

A list of de novo RNA editing candidates per sample was generated using REDItools,^10^ following the filtering procedure as described in Picardi and co-workes and Lo Giudice and co-workers.^11,12^ Differential RNA editing at detected sites was identified using REDITS selecting sites with a FDR correct Pvalue < 0.05.^13^

Recoding index (REI) was calculated using a custom python script working on REDItools tables. We considered as recoding sites all 1585 editing positions stored in REDIportal that are marked as non-synonymous in all three gene annotations available in the database.^14,15^ *Alu* editing index (AEI) was calculated using the methodology by Roth and co-workers. ^16^

RNA editing in long dsRNAs was investigated according to the methodology proposed by Barak and co-workers.^17^ For each gene annotated in RefSeq, BLAST was used to look for alignments of the sequence to itself (mature mRNA), keeping only matches with length > 40 and identity > 70%. BLAST matches involving the two strands (plus/minus hits) were considered putative dsRNAs. RNA editing at potential dsRNAs was detected by REDItools and summarized using an AEI-like metric. Differential RNA editing at putative dsRNAs was identified using the t-test, retaining only dsRNAs with an adjusted (Benjamini-Hochberg correction) p-value < 0.05.

Real-time RT-qPCR

MiRNA expression was validated by using miScript PCR system (Qiagen). cDNA was synthesized with miScript II RT Kit (Qiagen) following the manufacture instructions and about 0,6 ng was used as template in 25 μl qPCR reactions performed with miScript SYBR Green Kit (Qiagen). All the reactions were performed with biological triplicates using StepOnePlus instrument (Applied Biosystem). Reactions were incubated at 95°C for 15 min, then for 40 cycles at three step cycling (94°C for 15’’, 55°C for 30’’, 70°C for 30’’), and melting curves were used to confirm the specificity of each amplification product.

Primers: miScript Primer Assays specific for MIR221, MIR3614, MIR423, MIR3200, MIR3661, MIR1254, MIR320C1, MIR25, MIR151A (Qiagen).

RT-PCR

Total RNA was prepared from the cells using TRI reagent (Zymo Research, Irvine, CA, USA) according to manufacturer’s instruction followed by DNase treatment (DNase I, New England Biolabs inc.). cDNA was synthesized with M-MLV RT (Thermo Fisher Scientific) following the manufacturer’s instructions and used as template for PCR by using the MegaFi Fidelity Taq DNA Polymerase (ABM, Richmond, BC V6V 2J5, Canada).

Primers:

-MRPL30 FW: 5’-ATGGAGAAACCCCCATCTCT-3’; MRPL RW: 5’-AAACAATTTTGGCTGGGTACA-3’

-PAICS FW: 5’-ACCGTCTTCTCATCCTCTGT-3’; PAICS RW: 5’-AACGTGGGCCTTCAGAGAAC-3’

-FILAMIN B FW: 5’-CCAGTAAGGCCGAGATTACATT-3’; FILAMIN B RW: 5’-ATGAGGGATGAAGCGAACAG-3’

Immunofluorescence and confocal microscopy

LCLs were plated on a pre-sterilized coverslip in a 6-well plate at 20 x 10^4^ cells per well with 1× PBS. After 15-60 minutes cells were rinsed with PBS 1× and fixed with 4% paraformaldehyde for 15 minutes at room temperature (RT), followed by 3 washes with PBS 1×. Next the cells were permeabilized with 0.1% Triton X-100 in PBS 1× for 10 minutes at RT, and then incubated with a bovine serum albumin in PBS 1× containing (BSA) 1% supplemented with 0.05% Triton X-100 solution, for 1 hour at RT. Then the cells were incubated for 60 minutes at 37°C with the following primary antibodies: anti-ADAR1 (Santa Cruz Biotechnology, Dallas, TX, USA), anti-MDA5 (Thermo Fisher Scientific), anti-TIAR (Cell Signaling Technology), anti-NUCLEOLIN (Santa Cruz Biotechnology, Dallas, TX, USA). After 3 further washes with PBS 1×, the cells were stained with the secondary Alexa Fluor 488-labeled anti-rabbit antibody (Thermo Fisher Scientific) and the secondary Alexa Fluor 555-labeled anti-mouse antibody (Thermo Fisher Scientific) at room temperature for 60 minutes. After 3 washes with PBS 1×, the coverslip was mounted on a glass slide using one drop of dapi-mountant (Thermo Fisher Scientific).

HeLa cells were plated on a pre-sterilized coverslip in a 6-well plate at 60 × 10^3^ cells per well with complete media and transfected the next day with either pcDNA3.1 empty vector or pcDNA3.1 ADAR1 p150 wt or pADAR1 870 (expressing the Ala870Thr variant) or pADAR1 193 (expressing the Pro193Ala variant). Three days after transfection, cells were rinsed with 1× phosphate-buffered saline (PBS) and fixed with 4% paraformaldehyde for 20 minutes at room temperature (RT), followed by 3 washes with PBS 1×. Next the cells were permeabilized with 0.5% Triton X-100 and PBS 1× for 15 minutes at RT, and then incubated with 0.5% normal goat serum in PBS 1× for 60 minutes. For dual immunofluorescence staining, the cells were incubated with anti-ADAR1 antibody (Santa Cruz Biotechnology) and anti-TIAR antibody (Cell Signaling Technology) for 60 minutes at 37°C. After 3 further washes with PBS 1×, the cells were stained with the secondary Alexa Fluor 488-labeled anti-rabbit antibody (Thermo Fisher Scientific) and the secondary Alexa Fluor 555-labeled anti-mouse antibody (Thermo Fisher Scientific) at room temperature for 60 minutes. After 3 washes with PBS 1×, the coverslip was mounted on a glass slide using one drop of dapi-mountant (Thermo Fisher Scientific). Samples were analyzed as described for the LCLs.

UV Cross-Linking and Immunoprecipitation (CLIP)

LCLs were first rinsed in PBS 1× and then irradiated once with 400 mJ/cm^2^ at 254-nm UV using a crosslinker (UV Stratalinker 2400, Stratagene). After the crosslinking, cells were rinsed in PBS 1× and lysed in NP40 buffer supplemented with Protease inhibitors (Protease inhibitor cocktail 50×, Promega) and RNase Inhibitor (New England Biolabs inc.) for 20’ on ice. Two milligrams of cell extracts were pre-cleared on IgG/dynabeads protein G (Thermo Fisher Scientific) and then incubated for 1h at 4°C with rotation and then incubated for 3h or overnight at 4°C with either an anti-MDA5 (Thermo Fisher Scientific) or control IgG followed by incubation with dynabeads protein G (Thermo Fisher Scientific) for 1-3h at 4°C with rotation. After 3-4 washes with PBS 1×, the

beads and 10 μg of the total cell extract (input) were resolved by SDS-PAGE and transferred onto the nitrocellulose membrane and then analyzed by immunoblotting using specific antibodies.

Supplementary References

1. Liao Y, Smyth GK, Shi W. featureCounts: an efficient general-purpose program for assigning sequence reads to genomic features. Bioinformatics. 2014;30(7):923-930.
2. Love MI, Huber W, Anders S. Moderated estimation of fold change and dispersion for RNA-seq data with DESeq2. Genome Biol. 2014;15(12):550. https//www.doi.org/10.1186/s13059-014-0550-8.
3. Li Z, Zuo Y, Xu C, Varghese RS, et al. INDEED: R package for network based differential expression analysis. Proceedings (IEEE Int Conf Bioinformatics Biomed). 2018; 2018:2709-2712. https//www.doi/10.1109/BIBM.2018.8621426.
4. Langmead B, Trapnell C, Pop M, et al. Ultrafast and memory-efficient alignment of short DNA sequences to the human genome. Genome Biol. 2009;10(3): R25. https//www.doi.org/10.1186/gb-2009-10-3-r25.
5. Picardi E, Pesole G. REDItools: high-throughput RNA editing detection made easy. Bioinformatics. 2013;29(14):1813-1814.
6. Picardi E, Manzari C, Mastropasqua F, et al. Profiling RNA editing in human tissues: towards the inosinome Atlas. Sci Rep. 2015; 5:14941. https//www.doi/10.1038/srep14941
7. Lo Giudice C, Tangaro MA, Pesole G, Picardi E. Investigating RNA editing in deep transcriptome datasets with REDItools and REDIportal. Nat Protoc. 2020;15(3):1098-1131
8. Tran SS, Zhou Q, Xiao X. Statistical inference of differential RNA-editing sites from RNA-sequencing data by hierarchical modeling. Bioinformatics. 2020;36(9):2796-2804.
9. Picardi E, D'Erchia AM, Lo Giudice C, Pesole G. REDIportal: a comprehensive database of A-to-I RNA editing events in humans. Nucleic Acids Res. 2017;45(D1):D750-D757.
10. Mansi L, Tangaro MA, Lo Giudice C, et al. REDIportal: millions of novel A-to-I RNA editing events from thousands of RNAseq experiments. Nucleic Acids Res. 2021;49(D1):D1012-D1019.
11. Roth SH, Levanon EY, Eisenberg E. Genome-wide quantification of ADAR adenosine-to- inosine RNA editing activity. Nat Methods. 2019;16(11):1131-1138.
12. Barak M, Porath HT, Finkelstein G, et al. Purifying selection of long dsRNA is the first line of defense against false activation of innate immunity. Genome Biol.
